# On the Fourth Order Hamiltonian of an Asymmetric Rotor Molecule of Orthorhombic Symmetry

**DOI:** 10.6028/jres.067A.039

**Published:** 1963-08-01

**Authors:** Wm. B. Olson, H. C. Allen

## Abstract

The fourth order Hamiltonian of an asymmetric rotor molecule of orthorhombic symmetry given recently has been considerably reduced in complexity through the use of equations derived from the basic relationship among the angular momentum operators. The reduced Hamiltonian obtained provides a most convenient starting point for the calculation of rotational energy levels from a solution of the complete secular equation, for a perturbation theory solution to the problem of centrifugal distortion, and for the deduction of sum rules among the energy levels.

## 1. Introduction

Recently Chung and Parker [1] 1 have examined the general molecular vibrational-rotational Hamiltonian in the Goldsmith-Amat-Nielsen [2] formulation. They deduced Hamiltonians for asymmetric rotor molecules of orthorhombic, monoclinic, and triclinic point group symmetries and have included in their work all terms in *P*^4^ to the fourth order of approximation. No terms in *P*^6^ were included in their work, nor will they be included subsequently in this.

For the case of the asymmetric rotor Hamiltonian for molecules of the orthorhombic point groups (*C*_2_*_v_, V*, and *V_h_*) it is possible to reduce the Hamiltonian considerably and, by a redefinition of coefficients, to make valid a considerable body of previous work. It is our purpose to carry out these reductions, relate the results to previous work, and to provide a first order perturbation theory solution to the problem of centrifugal distortion, which, while entirely equivalent to that of Kivelson and Wilson [3], is in somewhat simpler form.

## 2. The Hamiltonian

We start with eq (17) of Chung and Parker in the molecular axis system of coordinates defined by the standard spectroscopic convention $I_{a}^{e}<I_{b}^{e}<I_{c}^{e}$. The following relations among the angular momentum operators that are useful for reducing the Hamiltonian may be derived from the commutation rules for the angular momentum operators.

$$P_{\alpha }^{2}P_{\beta }^{2}+P_{\beta }^{2}P_{\alpha }^{2}=P^{4}+P_{\gamma }^{4}-P_{\alpha }^{4}-P_{\beta }^{4}-2P^{2}P_{\gamma }^{2}$$ (1)

$$P_{\alpha }P_{\beta }P_{\alpha }P_{\beta }+P_{\beta }P_{\alpha }P_{\beta }P_{\alpha }=P^{4}+P_{\gamma }^{4}-P_{\alpha }^{4}-P_{\beta }^{4}-2P^{2}P_{\gamma }^{2}+\hbar^{2}(P_{\gamma }^{2}-P_{\alpha }^{2}-P_{\beta }^{2})$$ (2)

$$P_{\alpha }P_{\beta }^{2}P_{\alpha }=\frac{1}{2}[P^{4}+P_{\gamma }^{4}-P_{\alpha }^{4}-P_{\beta }^{4}-2P^{2}P_{\gamma }^{2}+2\hbar^{2}(P_{\gamma }^{2}-P_{\beta }^{2})]\;\alpha \neq \beta \neq \gamma =a,b,\;\text{or}\;c\;(\text{or}\;x,y,\;\text{or}\;z).$$ (3)

With the help of these relations we obtain a reduced Hamiltonian for molecules belonging to the orthorhombic point groups,

$$\begin{array}{r} H^{†}=h_{v}^{†*}+\sum_{\alpha }\alpha_{v}^{\prime }P_{\alpha }^{2}-\frac{1}{2}P^{2}\sum_{\alpha }\tau_{\beta \beta \gamma \gamma }^{v}P_{\alpha }^{2}+\frac{1}{4}\sum_{\alpha }\tau_{\alpha \alpha \beta \beta }^{v}P^{4}+\frac{1}{4}\sum_{\alpha }\left(\tau_{\alpha \alpha \alpha \alpha }^{v}+\tau_{\beta \beta \gamma \gamma }^{v}+\tau_{\alpha \alpha \beta \beta }^{v}-\tau_{\alpha \alpha \gamma \gamma }^{v}\right)P_{\alpha }^{4}\\ \alpha \neq \beta \neq \gamma =a,b,\;\text{and}\;c\;\text{in cyclic order}. \end{array}$$ (4)

In (4) the following definitions have been used for the constants

$$\alpha_{v}^{\prime }=\alpha_{v^{\prime }}+\frac{1}{4}\hbar^{2}\tau_{\alpha }^{v}$$ (5)

in which the *α_v_*_′_ are the A, B, and ℭ of Chung and Parker and where the *A, B*, and *C* of Chung and Parker are the reciprocal of twice the respective moments of inertia. Also:

$$\tau_{\alpha }^{v}=3\tau_{\beta \gamma \beta \gamma }+\rho_{\beta \gamma \beta \gamma }+\rho_{\beta \gamma \gamma \beta }+\rho_{\gamma \beta \beta \gamma }-2\tau_{\alpha \beta \alpha \beta }-\rho_{\alpha \beta \alpha \beta }-\rho_{\beta \alpha \alpha \beta }-2\tau_{\alpha \gamma \alpha \gamma }-\rho_{\alpha \gamma \alpha \gamma }-\rho_{\gamma \alpha \alpha \gamma }$$ (6)

$$\tau_{\alpha \alpha \alpha \alpha }^{v}=\tau_{\alpha \alpha \alpha \alpha }+\rho_{\alpha \alpha \alpha \alpha }$$ (7)

$$\tau_{\alpha \alpha \beta \beta }^{v\prime }=\tau_{\alpha \alpha \beta \beta }^{\prime }+2\tau_{\alpha \beta \alpha \beta }^{v\prime }$$ (8)

$$\tau_{\alpha \alpha \beta \beta }^{v\prime }=\tau_{\alpha \alpha \beta \beta }+\rho_{\alpha \alpha \beta \beta }$$ (9)

$$2\tau_{\alpha \beta \alpha \beta }^{v\prime }=2\tau_{\alpha \beta \alpha \beta }+\rho_{\alpha \beta \alpha \beta }+\frac{1}{2}\left(\rho_{\alpha \beta \beta \alpha }+\rho_{\beta \alpha \alpha \beta }\right).$$ (10)

In the preceding $h_{v}^{†*}$ and the *τ_αβγδ_* and *ρ_αβγδ_* are those of Chung and Parker, the *τ_αβγδ_* of Chung and Parker being the reciprocal of *ħ*^4^ times those of Kivelson and Wilson.

This convenient form of the Hamiltonian reveals that a constant similar to *D_J_* in the energy expression for linear, spherical and symmetric rotors may be defined, that is, a coefficient of *J*^2^(*J*+1)^2^ in the energy. Since this term is diagonal in *J*, *K*, *M* it may be factored out of the secular equation before its solution.

The cyclic nature of (4) indicates that this Hamiltonian may be solved using the systematic procedures introduced by King, Hainer, and Cross, [4] after extension of the latter to contain the matrix elements of the operators $P_{\alpha }^{4}$, by the choice of the most convenient representation, *I^r^* …. *III^l^*, defined by King, Hainer, and Cross (see table II, ref. 4).

Defining the effective rotational constants *A_v_, B_v_*, and *C_v_* in energy units as:

$$\alpha_{v}=\alpha_{v}^{\prime }\hbar^{2},$$ (11)

remembering that $P^{4}=P^{2}(P_{x}^{2}+P_{y}^{2}+P_{z}^{2})$, and using the definition:

$$\tau_{\alpha \alpha }^{v}=\tau_{\beta \beta \gamma \gamma }^{v}-\tau_{\gamma \gamma \alpha \alpha }^{v}-\tau_{\alpha \alpha \beta \beta }^{v},$$ (12)

where *α, β* and *γ* are taken as *a*, *b*, and *c* in cyclic order, we arrive at the following most convenient and explicit form for the vibrational-rotational part of the Hamiltonian:

$$\begin{array}{l} H=H^{†}-h_{v}^{†*}=[A_{v}/\hbar^{2}-J(J+1)(\hbar^{2}/4)\tau_{aa}^{v}]P_{a}^{2}+[B_{v}/\hbar^{2}-J(J+1)(\hbar^{2}/4)\tau_{bb}^{v}]P_{b}^{2}\\ \;+[C_{v}/\hbar^{2}-J(J+1)(\hbar^{2}/4)\tau_{cc}^{v}]P_{c}^{2}+\frac{1}{4}(\tau_{aaaa}^{v}+\tau_{aa}^{v})P_{a}^{4}\\ \;+\frac{1}{4}(\tau_{bbbb}^{v}+\tau_{bb}^{v})P_{b}^{4}+\frac{1}{4}(\tau_{cccc}^{v}+\tau_{cc}^{v})P_{c}^{4} \end{array}$$ (13)

In (13) no cross terms between the components of the angular momentum appear explicitly and the secular equation is readily set up using only the matrix elements of $P_{a}^{2}$, $P_{b}^{2}$, $P_{c}^{2}$, $P_{a}^{4}$, $P_{b}^{4}$ and $P_{c}^{4}$.

In order to make use of the systematization introduced by King, Hainer, and Cross the elements of $P_{x}^{4}$ and $P_{y}^{4}$ must be used in the same phase as they chose for *P_x_* and *P_y_.* Nowhere in the literature are these elements given using this phase. They are, however, readily deduced from those given by Wilson [5] since the only change required from his matrix elements is a reversal in sign of the *K*, *K*±2 elements of $P_{x}^{4}$ and $P_{y}^{4}$.

The coefficients of $P_{a}^{2}$, $P_{b}^{2}$, and $P_{c}^{2}$ can be regarded as effective inertial parameters with a *J* dependence in setting up the secular equation. Since the secular equation has no mixing between blocks of different *J*-factors, this observation can be used to reduce the work in solving the secular determinant. One merely uses a different set of effective inertial parameters for each *J*-factor.

Each *J*-factor can be further factored as in the case of the rigid asymmetric rotor into four factors that can be called *E*^±^ and *O*^±^ in complete analogy with the rigid case. These factors are conveniently written as:

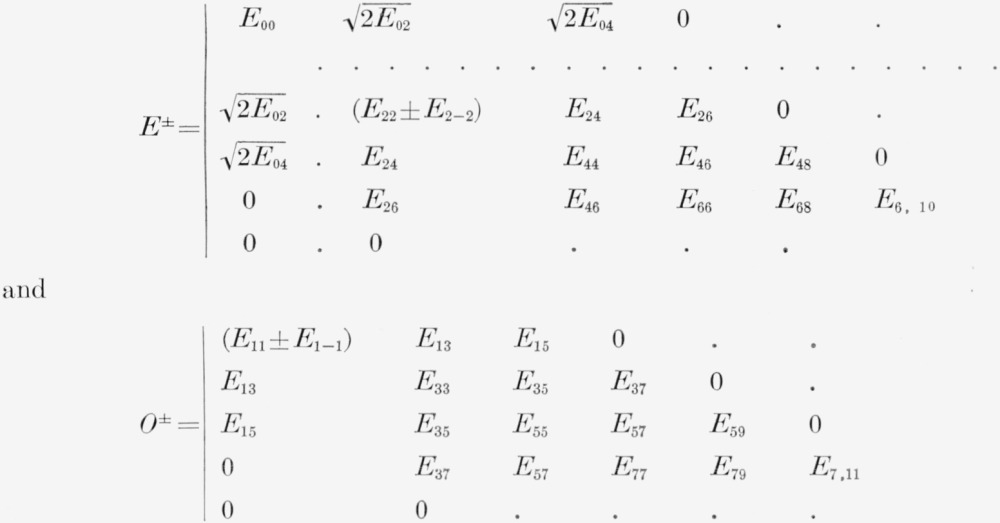
(14)where *E*^−^ is obtained from *E^+^* by removing the first row and column as indicated by the dots.

## 3. First Order Perturbation Theory

Equation (13) forms a convenient starting point for a perturbation treatment. In a first-order treatment the desired energies involve only the diagonal values of the perturbing operators. Consequently, eq (13) may be rewritten using only the average values of these operators in a representation in which the semirigid energy is diagonal,

$$E=E_{0}^{J}+\frac{1}{4}(\tau_{aaaa}^{v}+\tau_{aa}^{v})<P_{a}^{4}>+\frac{1}{4}(\tau_{bbbb}^{v}+\tau_{bb}^{r})<P_{b}^{4}>+\frac{1}{4}(\tau_{cccc}^{v}+\tau_{cc}^{v})<P_{c}^{4}>.$$ (15)

The values of $<P_{a}^{4}>$, $<P_{b}^{4}>$ and $<P_{c}^{4}>$ can be obtained from the work of Schwendeman [6].

For some purposes it may be convenient to use the symmetric rotor type centrifugal distortion constants given by Kivelson and Wilson rather than the $\tau_{\alpha \beta \gamma \delta }^{v}$. These centrifugal distortion constants of Kivelson and Wilson can be given accurate to the present order of approximation of the Hamiltonian by simply replacing the $\tau_{\alpha \beta \gamma \delta }^{\prime }$ of Kivelson and Wilson by $\tau_{\alpha \beta \gamma \delta }^{v}$.

## 4. Sum Rules

Recently sum rules for the energy levels of an orthorhombic asymmetric rotor have been published [7]. By the substitution mentioned above in a *I^r^* representation [4] these sum rules can be made valid to the present order of approximation. To obtain these sum rules substitute the right hand side of the following equations for the terms on the left hand side which appear in the sum rules.

$$\begin{array}{l} A=A_{v}\\ B=B_{v}\\ C=C_{v} \end{array}$$

R6−110DK=140[(τaaaav+τbbbbv+τccccv)+(τaav+τbbv+τccv)]−130(15DJ+5DJK+3DK)=140[(τaaaav+τbbbbv+τccccv)−23(τaav+τbbv+τccv)]DJ−2δJ−15DK=120[(τaaaav+τbbbbv−4τccccv)+(τaav+τbbv+τccv)]DJ+2δJ−15DK=120[(τaaaav+τccccv−4τbbbbv)+(τaav+τbbv+τccv)]DJ−R6−110DK=140[(τaaaav−4τbbbbv−4τccccv)+(τaav+τbbv+τccv)]6R6+15DK=140[(3τbbbbv+3τccccv−2τaaaav)+(3τbbv+3τccv+2τaav)]2(R5+R6+110DK)=140[(3τccccv−2τbbbbv−2τaaaav)+(3τccv−2τbbv−2τaav)]2(R5−R6−110DK)=140[(2τccccv+2τaaaav−3τbbbbv)+(2τccv+2τaav−3τbbv)]. (16)

## 5. Planar Orthorhombic Molecule

Dowling [8] and Oka and Morino [9] have given relations among the *τ_αβγδ_* of a planar orthorhombic molecule. Noting that the plane of the molecule must be the *ab* plane these become

$$\begin{array}{l} \tau_{bcbc}=\tau_{caca}=0\\ \tau_{bbbb}={\left(\frac{B_{e}}{C_{e}}\right)}^{2}\tau_{bbcc}-{\left(\frac{B_{e}}{A_{e}}\right)}^{2}\tau_{aabb}\\ \tau_{cccc}={\left(\frac{C_{e}}{B_{e}}\right)}^{2}\tau_{bbcc}+{\left(\frac{C_{e}}{A_{e}}\right)}^{2}\tau_{ccaa}\\ \tau_{aaaa}={\left(\frac{A_{e}}{C_{e}}\right)}^{2}\tau_{ccaa}-{\left(\frac{A_{e}}{B_{e}}\right)}^{2}\tau_{aabb}. \end{array}$$

As corresponding relations among the *ρ_αβγδ_* of the planar orthorhombic molecule have not yet been worked out, no special relations can yet be said to exist among the $\tau_{\alpha \beta \gamma \delta }^{v}$.

## 6. Conclusion

The Hamiltonian of an orthorhombic asymmetric rotor molecule, to the approximation considered in this work, has been set up in nine effective constants occurring as coefficients of six rotational operators.

This Hamiltonian appears to be in the simplest possible form, and to provide the most convenient starting point for any type of calculation concerning the rotation of this type of molecule.

## Footnotes

1 Figures in brackets indicate the literature references at the end of this paper.

## References

[1] Chung KT, Parker PM (1963). J Chem Phys.

[2] Goldsmith M, Amat G, Nielsen HH (1956). J Chem Phys.

[3] Kivelson D, Wilson EB (1952). J Chem Phys.

[4] King GW, Hainer RM, Cross PC (1943). J Chem Phys.

[5] Wilson EB (1937). J Chem Phys.

[b6-jresv67an4p359_a1b] 6R. H. Schwendeman, to be published.

[7] Allen HC, Olson WB (1962). J Chem Phys.

[8] Dowling Jerome M (1961). J Mol Spectroscopy.

[9] Oka T, Morino Y (1961). J Phys Soc Japan.

